# A novel cerclage wiring technique in intertrochanteric femoral fractures treated by intramedullary nails in young adults

**DOI:** 10.1186/s12891-018-2284-3

**Published:** 2018-10-06

**Authors:** You-Shui Gao, Yan-Jie Guo, Xin-Gang Yu, Yang Chen, Chen Chen, Nan-Ji Lu

**Affiliations:** Department of Orthopaedic Surgery, Shanghai Jiao Tong University Affiliated Sixth People’s Hospital, Shanghai, 200233 China

**Keywords:** Intertrochanteric femoral fractures, Cerclage augmentation, Intramedullary nail

## Abstract

**Background:**

Intertrochanteric femoral fractures (IFFs) in young adults, generally due to severe trauma, are increasingly presented. Different from IFFs in the geriatric population, these fractures in young adults are always comminuted and substantially displaced. Natural traction induced by musculature following IFFs determines closed reduction on a fracture table is extremely difficult.

**Methods:**

To achieve anatomical reduction before intramedullary nail (IMN) fixation, we made an extended or a mini petrotrochantetic incision to facilitate temporary reduction using a pointed clamp. Subsequently, a curved and cannulated wire-passer was employed to pass through a multistrand cable to surround displaced fragments and strengthen intertrochanteric fixation. Afterward, a standard procedure was conducted to nail the fracture.

**Results:**

We used the surgical technique in 9 young patients with an age range of 28~ 48 years old. The fractures were categorized as AO/OTA 31-A2.2 (3 cases) and 31-A2.3 (6 cases). The injury-to-surgery interval was 2.5 days on average. Mean operation time was 55 min. All fractures achieved anatomical reduction and healed within 14 weeks postoperatively without cable breakage, implant irritation or deep infection.

**Conclusions:**

In conclusion, the surrounding technique with cerclage wire in IFFs in young adults is an effective surgical technique with easily achieved anatomical reduction to facilitate operative maneuvers and fracture healing.

## Background

Intertrochanteric femoral fractures are frequent injuries in the elderly, mainly due to falling from a standing position [1]. Thankfully, these geriatric and osteoporotic femoral fractures can be fixed by intramedullary nails to facilitate an early leaving bed and support a desired functional recovery [2, 3]. In contrast, intertrochanteric femoral fractures in young adults are rarely seen and are mostly induced by high-energy injuries. Therefore, young intertrochanteric femoral fractures are comminuted and displaced at large. The muscular forces of the hip could deteriorate the fragment deformation, which makes anatomical reduction through closed maneuver quite difficult [4].

The story of subtrochanteric femoral fractures shows us that anatomical reduction through closed manipulation is of significant difficulty [4, 5]. To solve the problem, we can utilize techniques of assisted reduction and devices via a small lateral incision. Among these devices, various clamps and cerclage wire are frequently employed to achieve and maintain satisfactory reduction permanently or consistently [6–9].

**Fig. 1:**
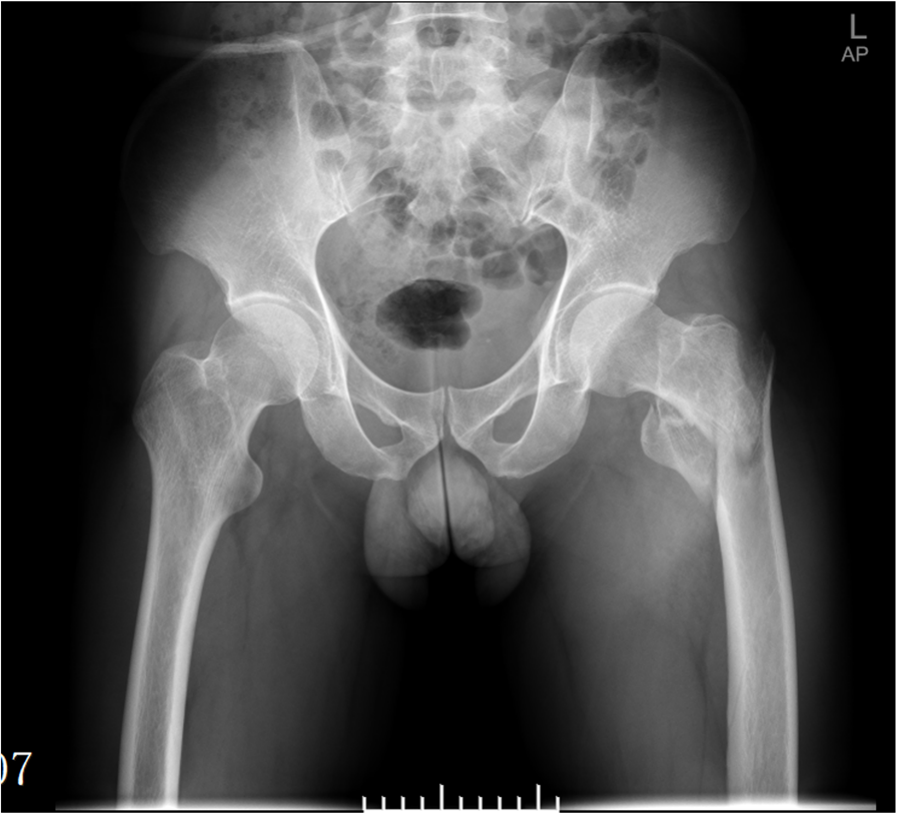
A 38-year-old man who fell from height and had a comminuted intertrochanteric femoral fracture, which was graded as AO/OTA 31-A2.3

In the current study, for comminuted and displaced intertrochanteric femoral fractures in young adults (Fig. 1), we made an extended or a mini lateral incision to palpate the displaced fragments. Afterward, a pointed reduction clamp was used to reposition the displaced fractures. Generally, the reduction could be easily maintained; however, loss of reduction could be encountered when reaming and with the entering of an intramedullary nail. Therefore, before making the starting point at the tip of the trochanter, we used a cerclage wire to assure the reduction for following manipulation of intramedullary nail fixation, while the displaced fragments could be surrounded by the wire. The novelty of our surgical technique is reflected in two aspects. First, the circumferential level was above the lesser trochanter and close to the posterior cortex. The quality of bone structure (the calcar) here is the best. Therefore, the augmentation by cerclage could be effective and rigid. Second, the wire we used also surrounded the displaced greater trochanter, which became stable following tightening of the wire. Thus, in the young adults, early partial weight bearing was encouraged immediately postoperatively. We will describe the surgical technique in detail and present pilot clinical results. We believe the surgical technique is competent for irreducible intertrochanteric femoral fractures in young adults.

## Methods

### Surgical techniques

Under general anesthesia, the patient is placed supine on a fracture table. The injured lower limb is stabilized in a traction boot while the uninvolved limb is placed in abduction to facilitate intraoperative imaging. Closed reduction maneuvers should be attempted under fluoroscopic control, generally through axial traction and adduction and internal rotation of the involved limb. Occasionally, elevation of the distal limb is helpful to achieve satisfactory reduction. Attempts for closed reduction should be made no more than 3 times. In case of failure reduction, methods of open reduction can be used on the same fracture table.

**Fig. 2:**
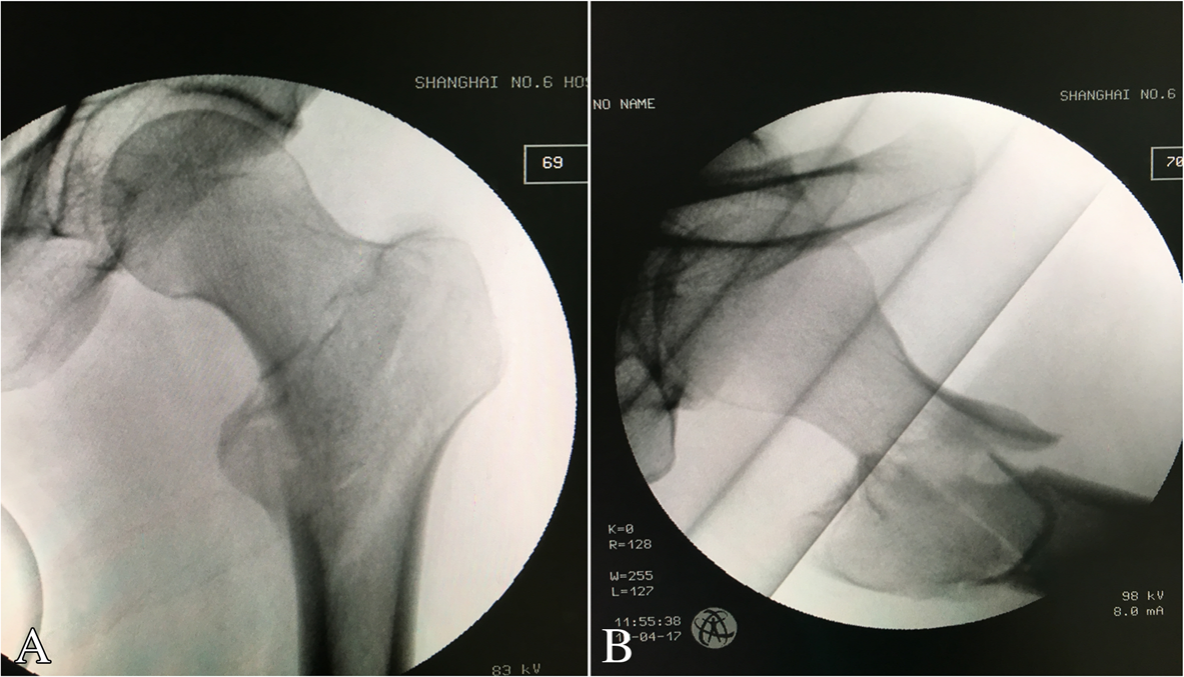
Attempt for closed reduction on a traction table. **a** Under axial traction, internal rotation and elevation of the distal limb; anteroposterior imaging shows the closed reduction is acceptable. **b** Lateral imaging shows the reduction is unsatisfactory. Displacement of proximal fragment is significant determined by muscular forces

**Fig. 3:**
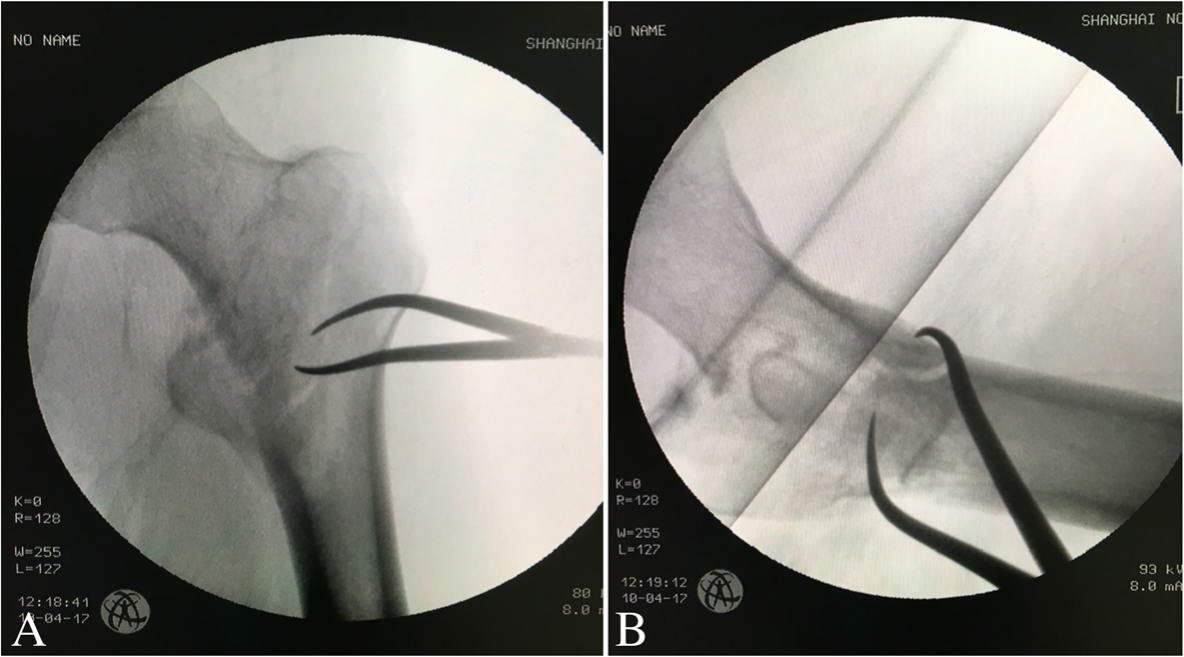
Open reduction with the help of a clamp. **a** A pointed reduction clamp is used to achieve anatomical reduction through a lateral incision. **b** The lateral view shows previous displacement is no longer in existence

**Fig. 4:**
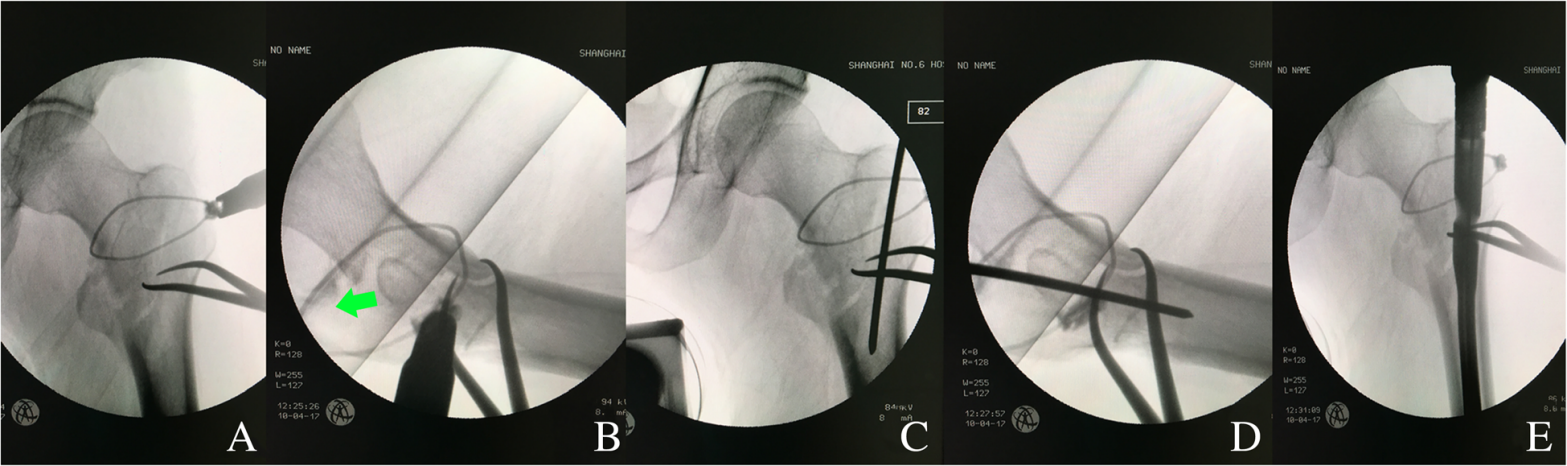
Cerclage wiring and entrance of intramedullary nail. **a** A circumferential wire is used to augment anatomical reduction. The wire surrounds the medial calcar above the fractured lesser trochanter and the lateral wall. **b** The lateral imaging shows the displaced fragment of the greater trochanter is also encircled (green arrow). Following entry of a guide pin from the tip of the greater trochanter, anteroposterior (**c**) and lateral (**d**) imaging show the reduction is maintained. **e** A proximal femoral nail antirotation (PFNA) is inserted to an appropriate depth

The limb is then prepared and draped, and the axial traction should be relaxed. The proximal incision above the greater trochanter for routine entrance of an intramedullary nail is extended for 4~ 6 cm, or a separated lateral incision 3~ 5 cm long is made. Alternatively, a separated mini lateral incision is made. The fascia lata is incised, and the fibers of the vastus lateralis are split bluntly along the anterior edge of the proximal femur. The muscular forces always make the proximal fragment displaced to the position of adduction, internal rotation and anterior flexion (Fig. 2). In the lateral incision, the fracture site can be palpated. Gentle traction is applied to make the fractured main fragments to approximately the same level. In this scenario, a pointed reduction clamp is helpful to achieve temporary anatomical reduction (Fig. 3). The smooth medial edge of the femur (calcar) can be palpated intraoperatively; meanwhile, the quality of reduction should be confirmed under fluoroscopy. To avoid reduction loss, we use a cerclage wire to augment reduction. A wire passer is inserted from anteromedial femur (above the lesser trochanter) to posterolateral position. In the case of multiple greater-trochanter fragments (always displaced to the posterior), they can be surrounded by the wire as well (Fig. 4). We believe the advantages of cerclage wire-augmented reduction are clear: 1. a cerclage wire can surround more displaced fragments, which are commonly seen in young intertrochanteric fractures; 2. a cerclage wire can free the reduction clamp to facilitate following operation if necessary; 3. reaming and insertion of an intramedullary nail might cause loss of reduction, which can be effectively prevented by the wire augmentation; and 4. a cerclage wire is beneficial to prevent breakage of the lateral wall. The quality of reduction is reconfirmed under fluoroscopy.

**Fig. 5:**
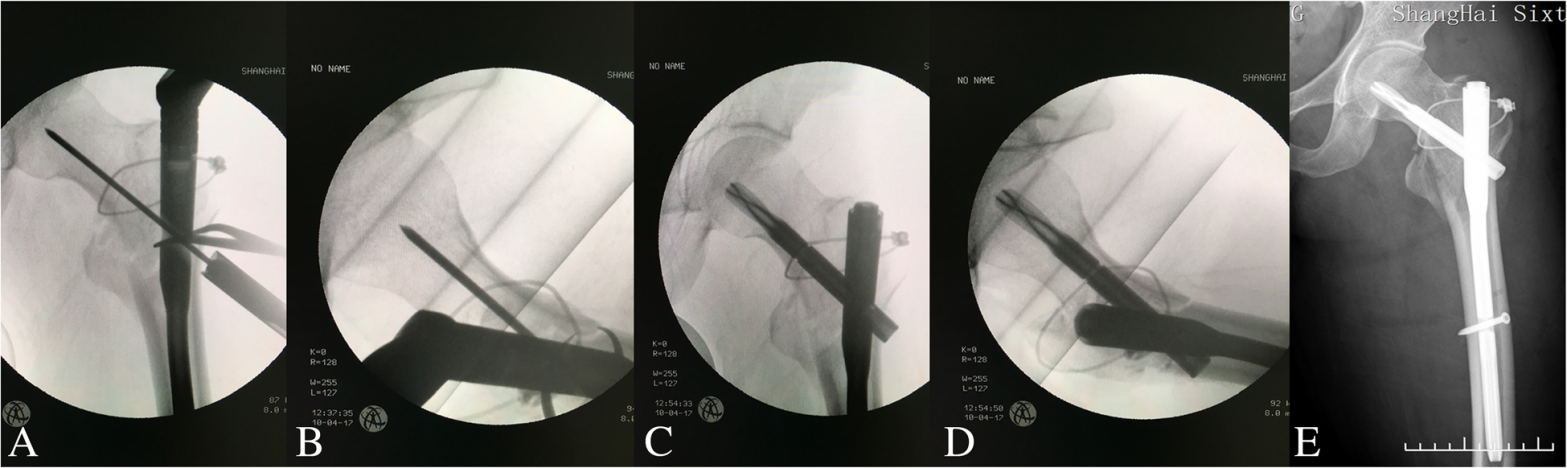
Routine maneuvers of nail fixation. Anteroposterior (**a**) and lateral (**b**) imaging show the guide pin for the blade. The pin should be in parallel with the axis of the femoral neck in both planes with correct tip-to-apex distance. Anteroposterior (**c**) and lateral (**d**) view shows anatomical reduction and satisfactory fixation by PFN-A. The wire is tightened, and the displaced fragments of the greater trochanter get a better position. **e** Postoperatively, anteroposterior radiograph shows the perfect combination of the wire and the nail

Afterward, a standard procedure of intramedullary fixation is performed. In brief, following the starting point at the tip of the great trochanter is confirmed under fluoroscopy, and the proximal femur is reamed along the guide pin. In the current study, a proximal femoral nail antirotation (PFNA) (Synthes) was the fixation of choice, mainly based on the surgeons’ preference. The nail is inserted to a proper depth, and a guide pin is drilled into the femoral head. It is important to obtain correct tip-to-apex distance (TAD, 5~ 10 mm) and anteversion angle (parallel to axial of the femoral neck). Afterwards, the screw blade is hammered into the femoral head and secured. Then the wire can be tightened to free the reduction clamp. We always find that the displaced fragments of the greater trochanter in posterior can achieve a better position on lateral view following clamp removal. Finally, the nail is stabilized with the static screw drilled. Usually, the end cap is used because hardware removal is preferable in young adults in China (Fig. 5).

### Patients

Between July 2016 and June 2017, we consecutively treated 9 young adults with comminuted and displaced intertrochanteric femoral fractures. All of the patients with such fractures underwent failure of closed reduction. Seven of the fractures were due to falling from height, and the other two were due to crash accidents. Eight patients were male, and one was female with an average age of 42.5 years (range, 28~ 48 years). According to AO/OTA classification, the fractures were categorized as 31-A2.2 in 3 cases and 31-A2.3 in 6 cases. The concomitant injuries included calcaneal fractures in 4 cases, wrist fractures in 2 and brain injuries in 2. The injury-to-surgery interval was 2.5 days (range, 2~ 5 days) on average.

Using the surgical techniques described above, we managed these intertrochanteric femoral fractures uneventfully. Calcaneal fractures in 3 and wrist fractures in 2 were treated surgically under the same anesthesia, following the surgical care of intertrochanteric femoral fractures. Other concomitant injuries were all treated conservatively. Mean operation time to manage intertrochanteric femoral fractures was 55 min (range, 45~ 70 min). Intraoperative blood loss was comparable to routine operations via closed reduction.

Postoperatively, anticoagulant drugs were prescribed to prevent deep vein thrombosis. We prefer to use low-molecular-weight heparin on hospitalization days and give patients Rivaroxaban or aspirin after discharge. Active exercises of the involved hip joint were initiated on the first postoperative day, while partial load bearing was determined on the patient’s general condition and concomitant injuries. The study has been approved by the ethics committee of Shanghai Sixth People’s Hospital.

## Results

All patients achieved anatomical reduction and underwent routine follow-up until fracture healing. There was no early complication, such as infection, or late complication, such as delayed union, nonunion, implant breakage or cutoff. The fracture had obvious callus formation within 14 weeks. Afterward, patients were encouraged to perform full weight bearing. The patients returned to their previous position with a total treatment duration of 16 weeks on average (range, 14~ 20 weeks).

## Discussion

Anatomical reduction is of critical importance in young intertrochanteric femoral fractures. Surgical maneuvers require satisfactory reduction achieved [10–14]. It is always highlighted to not ream an unreduced fracture [15]. Otherwise, intraoperative complications, including perforation and dehiscence of the femur, deterioration of fracture dislocation, and neurovascular damage, might be encountered. Anatomical reduction is also the prominent determinant of fracture healing and functional recovery.

To achieve satisfactory reduction, many assistant surgical techniques have been attempted for irreducible and unstable intertrochanteric fractures. In brief, a mini incision is always made as the pathway to palpate the displaced fracture. The lateral musculature can be bluntly detached to adapt the reduction devices. Previous studies recommended reduction clamp, pointed reduction clamp, bone hooks and bucking bar to obtain reduction [7, 16]. However, the reduction is not easy to be maintained during reaming and nail fixation. Alternatively, the surgeon has to be exposed under fluoroscopy radiation. Undoubtedly, loosening of reduction device before reaming or nail insertion could result in loss of reduction and delay of operation.

Cerclage wiring, a simple and historical surgical technique, has been used in fracture fixation solely or in combination with other implants, and gets its popularity in arthroplasty [17–28]. The concern with cerclage is the potential disruption to regional blood circulation, consequently delaying the fracture healing. The frustration might also include more operation time, cost and bleeding [29]. An animal study showed cable cerclage around the femoral cortex significantly decreased blood circulation in the area [30]. However, Apivatthakakul and colleagues found that percutaneous cerclage wiring resulted in minimal disruption of the blood supply of the femur through cadaveric injection study [31]. The experiment on cerclage-bone interface mechanics revealed cerclage provided a point contact fixation on the femoral shaft with blood-supply preserving principle [32]. Clinically, plenty of recent studies confirmed the beneficial effects of cerclage wiring in the care of peritrochanteric femoral fractures as well as in humeral shaft fractures and acetabular fractures. It seems that a cerclage wire facilitates fracture reduction and does not interrupt fracture healing.

To cerclage or not might become an ongoing debate. Nevertheless, the benefits and potential detriments of a wire should be noted for young surgeons. Closed reduction and intramedullary nail fixation has become the treatment of choice for unstable intertrochanteric femoral fractures [33]. To minimize detrimental effects, one can use temporary cerclage wiring technique alternatively. It should be highlighted that reduction was obtained with clamps and maintained with a wire in the current study. Wire should not act in a reduction role because the increased compression during tightening might induce iatrogenic fractures. The wire can also secure posterior fragments. The lateral wall of the trochanter is protected by a wire, which has been widely used in revision of hip arthroplasty.

The limitations of the current study should be noted as well. The number of treated cases is small due to the rarity of intertrochanteric fractures in young adults. Although all fractures healed in the follow-up, the implant is not removed to date, and long-term results have to be observed. In our early practice, we only maintained the reduction with a clamp and never used a wire temporarily or permanently. However, the clamp might become loosened when reaming. Although surgeons might prefer their familiar techniques, we believe cerclage wiring is simple and direct in the care of difficult intertrochanteric fractures.

## Conclusions

Cerclage wiring is a beneficial surgical technique in the care of young intertrochanteric femoral fractures that are irreducible through closed maneuvers. The wire is helpful to maintain the reduced fractures, to facilitate reaming and nailing, to reduce intraoperative complications and not to hinder fracture healing.
